# Differential regulation drives plasticity in sex determination gene networks

**DOI:** 10.1186/1471-2148-10-388

**Published:** 2010-12-16

**Authors:** Thomas MacCarthy, Robert M Seymour, Andrew Pomiankowski

**Affiliations:** 1 CoMPLEX, University College London, Gower Street, London WC1E 6BT, UK; 2 Department of Systems and Computational Biology, Albert Einstein College of Medicine, Bronx, NY 10461, USA; 3 Department of Mathematics, University College London, Gower Street, London WC1E 6BT, UK; 4 The Galton Laboratory, Research Department of Genetics, Evolution and Environment, University College London, 4 Stephenson Way, London NW1 2HE, UK

## Abstract

**Background:**

Sex determination networks evolve rapidly and have been studied intensely across many species, particularly in insects, thus presenting good models to study the evolutionary plasticity of gene networks.

**Results:**

We study the evolution of an unlinked gene capable of regulating an existing diploid sex determination system. Differential gene expression determines phenotypic sex and fitness, dramatically reducing the number of assumptions of previous models. It allows us to make a quantitative evaluation of the full range of evolutionary outcomes of the system and an assessment of the likely contribution of sexual conflict to change in sex determination systems. Our results show under what conditions network mutations causing differential regulation can lead to the reshaping of sex determination networks.

**Conclusion:**

The analysis demonstrates the complex relationship between mutation and outcome: the same mutation can produce many different evolved populations, while the same evolved population can be produced by many different mutations. Existing network structure alters the constraints and frequency of evolutionary changes, which include the recruitment of new regulators, changes in heterogamety, protected polymorphisms, and transitions to a new locus that controls sex determination.

## Background

Differential regulation of gene expression plays a primary role in the evolution of developmental networks [1,2]. In recent years there has been considerable progress towards understanding the evolution of gene regulation in development [3-5]. In some pathways, adaptive mutations appear constrained to central hub positions, as with the shavenbaby (*ovo*) locus for insect trichome differentiation [6,7], or bric-a-brac (*bab*) for fly abdominal pigmentation [8]. In contrast, other developmental processes allow adaptive mutations to occur throughout the network, as with *Drosophila *wing pigmentation [9,10] and wing polyphenism in ants [11]. Although progress in the field is advancing rapidly, the understanding of the evolutionary mechanisms driving regulatory changes is still limited. To address this issue we consider sex determination networks, as they evolve rapidly and are well characterized in species at varying phylogenetic distances, for example insects [12,13], worms [14], fishes [15] and mammals [16]. Sex determination is also a relatively simple phenotypic task compared to many other developmental functions such as somite formation or morphogenesis where complex pattern formation is required.

Comparative study shows that sex determination networks evolve by recruitment of novel regulators higher up the pathway [17]. For example, the gene *Sex-lethal *(*Sxl*), an upstream regulator of sex determination first found in *D. melanogaster *[18], does not appear to play any role in sex determination outside the Drosophilidae [13,19,20]. In contrast, the downstream regulator *doublesex *(*dsx*) is conserved across insects, worms [21,22] and mammals [23]. A similar paradigm has been proposed for segmentation in arthropods where segment-polarity genes such as engrailed (*en*) appear highly conserved and where the degree of conservation becomes progressively reduced for genes both upstream and downstream [24,25]. In sex determination, the addition of upstream elements results in considerable change in the regulatory role of downstream elements. For example, the gene *transformer *(*tra*) autoregulates in the Mediterranean fruit fly *Ceratitis capitata *[26] but lost this capacity when *Sxl *was recruited as an upstream regulator in *Drosophila*. Likewise, high regulatory variability is seen in the phylogenetically common *dsx *locus, which is regulated via alternative splicing of RNA by *tra *in many Diptera [13], but via the unrelated transcription factor *tra-1 *in worms [14].

Previous theoretical study showed how regulatory changes causing alterations in signal strength, could account for the evolution of the sex determination regulatory cascade of *D. melanogaster *in a step-by-step fashion [27]. In seeking to explain the specific case of *D. melanogaster*, particular evolutionary outcomes were addressed to account for the peculiarities of the genes involved in the network, for example multiple promoter sites, alternative splicing, and premature stop codons [27]. This detailed approach can be contrasted with others that have considered the population genetic conditions which must be met to allow the spread of autosomal modifiers of sex determination factors located on the sex chromosomes [28-30]. This has allowed sex ratio imbalance [28] and sexual antagonism [30] to be investigated as likely causes of change in sex determination. But research taking the latter approach has neglected to explicitly model the network dynamics and thus cannot explain how particular network architectures have arisen.

Here we present a model that combines gene network evolution with population genetics, allowing us to study the effect of differential gene expression with general applicability and minimal assumptions. Standard approaches to gene network evolution have used haploid models to simplify the dynamics [31-38]. This is not possible here as gene dosage is a key aspect in the sex determination of higher organisms. We consider how an unlinked gene, not previously involved in sex determination, can be recruited to regulate an existing diploid sex determination system. We assume that the ancestral system is controlled by polymorphism at a single sex-determining locus. The regulatory changes caused by the recruited gene can bring about a new sex determining locus, induce heterogamety changes or stabilize multiple male or female genotypes (protected polymorphisms). Furthermore, the same mutation can produce many different evolved outcomes, while the same evolved outcome can be produced by many different mutations. In the next section we define the model and follow this with a specific application to sex determination; further details of the simulations are given in the Methods.

## General Model

We extend a general model of gene regulation [38,39], using a network-oriented definition of alleles. First, each allele *i *may be regulated by an allele *j *at another locus (e.g. via *cis*-regulatory elements or RNA splicing sites), indicated by a parameter *I_i, j _*∈ {-1, 0, 1} which is 1 for up-regulation, -1 for down-regulation and 0 for no regulation. Second, an allele *i *may code for a regulatory domain (e.g. transcription factor or RNA recognition motif), indicated by a parameter *Z_i _*∈ {0, 1} which is 1 when the allele is regulatory and 0 otherwise. Third, each allele *i *has a constitutive output level defined by a parameter *T_i_*, which is either low (*T_i _*= -1) or high (*T_i _*= 1). These features determine the (time-dependent) expression level *S_i _*of each allele *i *via the dynamical system

(1) $$\frac{dS_{i}}{dt}=\sigma ([\sum_{j}I_{i,j}Z_{j}S_{j}]+kT_{i})-S_{i}.$$

The summation is over all alleles *j*, and we assume that

(2) $$\sigma (x)=\frac{1}{1+e^{-hx}}$$

is a sigmoid function. Most gene regulatory interactions can be approximated using this class of function [40-42] which varies between 0 and 1 with maximum steepness *h *(see Table 1 for full list of symbols). The initial conditions are *S_i_*(0) = *σ*(*kT_i_*) which are equilibrium expression levels if there is no regulation. The parameter *k *> 0 is a global constant that defines the relative contribution of the constitutive output to total gene expression. Note that the steady state output ${\hat{S}}_{i}$ of allele *i *is the solution of

**Table 1:** List of symbols

Symbol	Description
*I_i, j_*	regulation of allele *i *by *j*, which can take values -1, 0, +1
*Z_i_*	allele *i *is capable of regulation if *Z_i _*= 1, or not if *Z_i _*= 0
*T_i_*	constitutive expression of allele *i *is low (*T_i _*= -1) or high (*T_i _*= +1)
*k*	positive constant affecting constitutive expression level
*σ*(*x*)	sigmoid function varying between 0 and 1
*h*	steepness of sigmoid function *σ*(*x*)
D locus	expression level at D locus controls sex determination and fitness
m, f	ancestral alleles at D locus
m^+^, f^+^, m^-^, f^-^	mutant alleles of m and f allowing positive (^+^) or negative (^-^) regulation
R locus	regulator of D locus
a	ancestral allele at R locus
A	mutant allele at R locus, capable of regulating D locus alleles
${\hat{S}}_{\text{D}}$	steady state expression of both alleles at D locus
$\Delta {\hat{S}}_{\text{D}}$	D locus expression difference (evolved minus ancestral)
*θ*	sex determination threshold; if ${\hat{S}}_{\text{D}}>\theta$ then female, otherwise male.
*w*_M_, *w*_F_	selection on males and females respectively
*W*_M_, *W*_F_	fitness of male and female respectively

(3) $$S_{i}=\sigma ([\sum_{j}I_{i,j}Z_{j}S_{j}]+kT_{i})$$

attained from these initial conditions. Note that this model can also incorporate autoregulation by allowing alleles at the same locus to regulate their own expression (i.e. including alleles *i *in the summation term of Eq(1)).

## Model of Sex Determination

In our application of this general model to sex determination, the ancestral sex determination system is assumed to be controlled by a single locus D segregating for two alleles m and f. As there is no regulation in the ancestral condition, Eq(3) simplifies to

(4) $${\hat{S}}_{i}=\sigma (kT_{i}).$$

The two alleles m and f are assumed to have constitutive low (*T*_m _= -1) and high (*T*_f _= + 1) output levels respectively. We consider an XY male heterogametic system consisting of m/f males and f/f females (ancestral female heterozygosity is symmetric to the male heterozygous case, see Additional file 1).

The D locus controls sex determination. Specifically, if the sum of outputs of the two D locus alleles, d1 and d2, exceeds a threshold *θ*, ${\hat{S}}_{\text{D}}={\hat{S}}_{\text{d1}}+{\hat{S}}_{\text{d2}}>\theta$, then the phenotype is female, otherwise it is male. This system is inspired by the way doublesex (*dsx*) expression determines somatic sex in *Drosophila*, where high expression of the female form of the *dsx *protein is required for a female phenotype [12]. In our model, other genetic loci do not affect sex determination directly, though they can do so indirectly by altering gene expression at the D locus. Given ancestral male and female expression levels ${\hat{S}}_{\text{DM}}$ and ${\hat{S}}_{\text{DF}}$, respectively, we set $\theta =({\hat{S}}_{\text{DM}}+{\hat{S}}_{\text{DF}})/2$. In the ancestral system, we assume that there are no regulatory interactions controlling the D locus. Hence male expression is ${\hat{S}}_{\text{DM}}={\hat{S}}_{\text{m}}+{\hat{S}}_{\text{f}}=\sigma (k)+\sigma (-k)=1$ and ${\hat{S}}_{\text{DF}}=2{\hat{S}}_{\text{f}}=2\sigma (k)$. Therefore, *θ *= *σ *(*k*)+1/2. Thus, larger values of *k *mean a larger difference in gene expression around the threshold value *θ *between the two sexes.

We study the evolution of regulation controlled by a second locus R. We start from a situation in which the R locus is fixed for an allele a that does not regulate the D locus, *Z*_a _= 0. Consider a mutant allele A at the R locus that has a regulatory domain able to interact with the D locus, *Z*_A _= 1. In order for a regulatory interaction to evolve, the R locus allele A must be present together with a variant m or f allele at the D locus that is capable of being regulated by A. The m and f variant alleles are labeled according to whether the interaction causes up-regulation of expression (m^+^, f^+ ^with $I_{{\text{m}}^{+}\text{, A}}=I_{{\text{f}}^{+}\text{, A}}=1$) or down-regulation of expression (m^-^, f^- ^with $I_{{\text{m}}^{\text{-}}\text{, A}}=I_{{\text{f}}^{\text{-}}\text{, A}}=-1$). We use Eq(4) assuming *T*_A _*= *1 (i.e. high constitutive expression, see Methods) to calculate the expression level of the alleles at the R locus, and then use Eq(3) to calculate the regulated expression of the alleles at the D locus.

As the evolution of regulation by the R locus of the D locus requires change at both loci, we consider that one part of the connection exists in the background state, with the remaining part being completed by a subsequent mutation at the other locus. For example, if the allele A (R locus) is already present in the background state, it can complete a connection through the mutation of the f allele to the f^+ ^allele (D locus) which contains a novel input binding domain. This creates a positive regulatory connection from the R locus to the D locus. We denote this example a→A/f→f^+ ^(background/mutant). If we reverse the order to f→f^+^/a→A, then the allele f^+ ^already exists in the background state and the connection is completed by the mutation of the a allele to the A allele which contains a novel output regulatory domain.

There are eight possible combinations of background state and mutant that give rise to a novel regulatory connection: (1) a→A/f→f^-^, (2) a→A/f→f^+^, (3) a→A/m→m^-^, (4) a→A/m→m^+^, (5) f→f^-^/a→A, (6) f→f^+^/a→A, (7) m→m^-^/a→A, (8) m→m^+^/a→A (Table 2). Note that in cases (1) to (4), the background state allele A is assumed to be at fixation, and the consequence of introducing a mutant at the D locus is considered (either f^-^, f^+^, m^- ^or m^+^). In cases (5) to (8), either f^-^, f^+^, m^- ^or m^+ ^already are present in the background state (having replaced f or m), and we follow the mutant A allele introduced at the R locus. Note that the first four cases involve a *cis *mutation at the D locus occurring after a *trans *mutation at the R locus, and *vice versa *for the second four cases.

**Table 2:** Mutations and corresponding genotypes

**Mutation pair**^**a**^	Ancestral male	Ancestral female	Mutant
a→A/f→f^-^	A/A;m/f	A/A;f/f	A/A;m/f^-^
a→A/f→f^+^			A/A;m/f^+^
a→A/m→m^-^			A/A;m^-^/f
a→A/m→m^+^			A/A;m^+^/f
			
f→f^-^/a→A	a/a;m/f^-^	a/a;f^-^/f^-^	a/A;m/f^-^
f→f^+^/a→A	a/a;m/f^+^	a/a;f^+^/f^+^	a/A;m/f^+^
m→m^-^/a→A	a/a;m^-^/f	a/a;f/f	a/A;m^-^/f
m→m^+^/a→A	a/a;m^+^/f	a/a;f/f	a/A;m^+^/f

a: Each mutation pair is shown in the form background/mutant.

The new regulatory interaction between the R and D loci alters ${\hat{S}}_{\text{D}}$. This can cause a change in phenotypic sex. In addition, it can cause a change in the fitness (*W*) of each genotype. In the background state, male (*W*_M_) and female (*W*_F_) fitness are set to 1. The fitness of mutant genotypes is measured relative to the background state genotypes: $W_{\text{F}}=1+\Delta {\hat{S}}_{\text{DF}}w_{\text{F}}$ if the novel genotype is female, and $W_{\text{M}}=1+\Delta {\hat{S}}_{\text{DM}}w_{\text{M}}$ if it is male, where $\Delta {\hat{S}}_{\text{D}}$ is the difference in D locus expression caused by the mutant relative to the unregulated ancestral genotype (i.e. evolved minus ancestral gene expression). For example, consider a mutant male with genotype a/A;m^+^/f and expression in males ${\hat{S}}_{\text{DM}}=\sigma (\sigma (k)-k)+\sigma (k)$ (given we assume *T*_A _= 1 - see Methods). If the ancestral male was a/a; m^+^/f then the ancestral expression is ${\hat{S}}_{\text{DM}}=\sigma (-k)+\sigma (k)$, and the difference is $\Delta {\hat{S}}_{\text{D}}=\sigma (\sigma (k)-k)-\sigma (-k)$. The fitness parameters *w*_M _and *w*_F _define the direction and magnitude of selection in males and females respectively. Within the simulations (see Methods), we use random numbers in the range (-1/2,1/2) for both *W*_F _and *W*_M _to avoid negative fitness values for *W*_F _and *W*_M _(as the range for $\Delta {\hat{S}}_{\text{D}}$ is -2 to +2). We assume that fitness is not directly affected by the R locus, although change at this locus may of course affect fitness indirectly via ${\hat{S}}_{\text{D}}$. In the next section we use an example to illustrate how the model functions.

## Results and Discussion

We used simulations within a standard population-genetic framework to study the fate of pairs of mutations that establish new regulatory connections (see Methods). For each run, we recorded whether the mutation spread and caused a ***transition ***to the new sex determining R locus, or ***recruitment ***of the mutant with sex determination remaining at the ancestral D locus. We also noted whether transitions and recruitments caused a change in ***heterogamety***. A number of simulations resulted in ***protected polymorphisms ***in which a stable equilibrium was reached that contained more than one male or female genotype. We now discuss the frequency of these outcomes.

### The f→f^-^/a→A mutation pair

**Figure 1:**
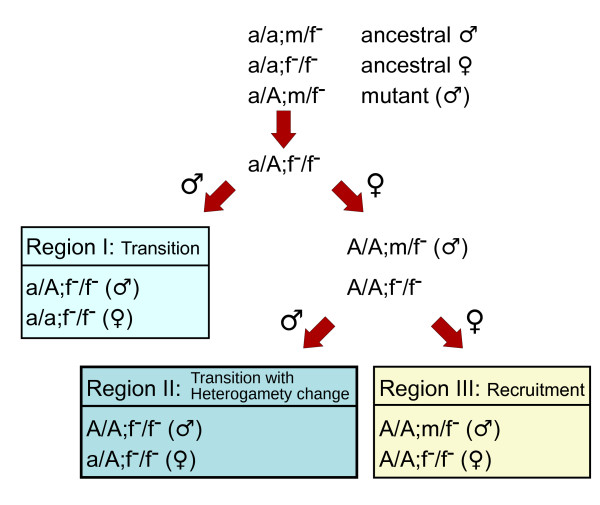
**Evolution of mutation pair f→f^-^/a→A**. The mutant A is introduced at low frequency in the genotype a/A;m/f^-^. Through interbreeding, a range of other genotypes are produced in subsequent generations (represented by arrows). Phenotypic sex (i.e. indicated above the arrows) of these novel genotypes depends on the values of *k *and *h *(see Figure 2(e)). Three potential outcomes are possible: transition to a new sex determining locus (Region I), transition coupled with a change in heterogamety (Region II), or recruitment of allele A without change in the sex determining locus (Region III).

**Figure 2:**
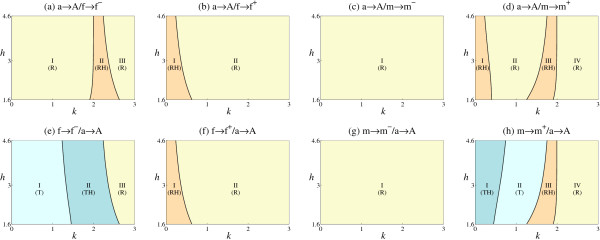
**Evolutionary outcomes**. Evolutionary outcomes depend heavily on *k *(the level of constitutive expression across both loci) and *h *(the steepness of the sigmoid function σ of Eq (2)) which jointly determine the change in gene expression at the D locus (${\hat{S}}_{D}$) caused by the A regulatory allele at the R locus. For each mutation pair, we map regions in which there are transitions in sex determination locus (T), recruitment without transition (R), and changes in heterogamety (H).

To illustrate our approach, we consider the mutation pair f→f^-^/a→A (Figure 1). In this case the ancestral male and female genotypes are m/f^- ^and f^-^/f^- ^respectively (with the a allele at fixation). The mutant A is then introduced at low frequency. The A mutant down-regulates f^-^, so the expression of ${\hat{S}}_{\text{D}}$ is lower in a/A;m/f^- ^males than in ancestral males. The A mutant also reduces ${\hat{S}}_{\text{D}}$ in the a/A;f^-^/f^- ^genotype. If ${\hat{S}}_{\text{D}}-\theta =2\sigma (-\sigma (k)+k)-(\sigma (k)+1/2)<0$, this genotype is transformed into a male and the sex determination system can undergo a transition from the D to the R locus. The m allele is lost resulting in heterozygous a/A;f^-^/f^- ^males and homozygous a/a;f^-^/f^- ^females. There is no change in the female genotype, but the dominant masculinizing m allele at the D locus is replaced by the dominant masculinizing A allele at the R locus. This occurs in Region I of Figure 2(e).

In contrast, if the genotype a/A;f^-^/f^- ^is female (Figure 1), matings between this female and mutant males (a/A;m/f^-^) produces two new genotypes that are AA homozygous: A/A;m/f^- ^and A/A;f^-^/f^-^. The genotype A/A;m/f^- ^is male because its ${\hat{S}}_{\text{D}}$ is lower than in the ancestral male. The genotype A/A;f^-^/f^- ^can be male or female. If 2*σ*(-2(*k*)+*k*)-(*σ*(*k*)+1/2) < 0 then it is male, which can lead to a population composed of A/A;f^-^/f^- ^males and a/A;f^-^/f^- ^females (Region II, Figure 2(e)). Once again there is a transition of control of sex determination from the D to the R locus, but in this case with an attendant change in heterogamety. The A mutant is a recessive masculinizer which replaces the m allele at the D locus. In contrast, if the genotype A/A;f^-^/f^- ^is female, this can lead to a population of A/A;m/f^- ^males and A/A;f^-^/f^- ^females. Here, the mutant allele A is recruited, replacing the ancestral allele a, without changing either the sex determination locus or heterogamety.

This example shows how multiple outcomes follow from a single mutation a→A which causes the network to down-regulate the pre-existing f^- ^allele. The A mutant can cause a transition in the sex determination system, with or without change in heterogamety or just simple recruitment of A without any change in the sex determining system (Table 3).

**Table 3:** Simulation results for all mutations

			Evolved Genotype		**Frequency of Outcome**^**a**^					
				
Mutation pair	Region	Outcome^b^	Male	Female	*W*_M_* > 1* *W*_F _*= 1*	*W*_M _*= 1* *W*_F_* > 1*	*W*_M_*>1* *W*_F_* > 1*	*W*_M_* > 1* *W*_F_* < 1*	*W*_M_* < 1* *W*_F_* > 1*	*Protected polymorphism*
a→A/f→f^-^	I	R	f/f^-^	f/f	3227	0	0	0	0	0
	II	RH	f^-^/f^-^	f/f^-^	0	0	246	0	1	407
	III	R	m/f^-^	f^-^/f^-^	0	0	516	130	420	12
										
a→A/f→f^+^	I	RH	m/m	m/f^+^	0	0	140	69	4	395
	II	R	m/f^+^	f^+^/f^+^	0	0	2174	507	1637	22
										
a→A/m→m^-^	I	R	m^-^/f	f/f	5028	0	0	0	0	0
										
a→A/m→m^+^	I	RH	m/m	m/m^+^	0	0	133	50	1	336
	II	R	m/m^+^	m^+^/m^+^	0	0	1065	246	779	15
	III	RH	m^+^/m^+^	m^+^/f	0	0	231	0	1	438
	IV	R	m^+^/f	f/f	1705	0	0	0	0	0
										
f→f^-^/a→A	I	T	a/A;f^-^/f^-^	a/a;f^-^/f^-^	2163	0	0	0	0	0
	II	TH	A/A;f^-^/f^-^	a/A;f^-^/f^-^	0	0	682	122	30	955
	III	R	A/A;m/f^-^	A/A;f^-^/f^-^	0	0	540	115	374	22
										
f→f^+^/a→A	I	RH	A/A;m/m	A/A;m/f^+^	0	0	190	87	48	321
	II	R	A/A;m/f^+^	A/A;f^+^/f^+^	0	0	2226	505	1622	32
										
m→m^-^/a→A	I	R	A/A;m^-^/f	A/A;f/f	5063	0	0	0	0	0
										
m→m^+^/a→A	I	TH	a/a;m^+^/m^+^	a/A;m^+^/m^+^	0	0	493	9	132	344
	II	T	a/A;m^+^/m^+^	A/A;m^+^/m^+^	0	0	19	13	25	1491
	III	RH	A/A;m^+^/m^+^	A/A;m^+^/f	0	0	298	20	2	338
	IV	R	A/A;m^+^/f	A/A;f/f	1741	0	0	0	0	0

a: Given 10,000 independent simulations, outcomes for each mutation pair are separated into one or more regions in parameter space (see Figure 2), in addition, the frequency of protected polymorphisms is given for each region (see Figure 4).

b: Outcomes are coded as transitions (T) or recruitments (R), with change in heterogamety (H).

The outcomes depend on the values of *k *and *h *that determine the change in gene expression at the D locus (${\hat{S}}_{D}$) due to the novel regulatory connection of the A allele at the R locus. The parameter *k *determines the background level of constitutive expression across both loci. It has a major effect on the evolutionary outcome (Figure 2). When *k *is high, regulation by A is relatively weak and thus major re-organisation of the sex determination systems do not occur. In contrast when *k *is low, regulation by A is relatively strong, allowing changes in genotypic sex, changes in heterogamety and transitions of the sex determination system to the R locus. The parameter *h *determines the steepness of the sigmoid function *σ *of Eq (2), and has a minor effect on the evolutionary outcome (Figure 2). This reflects the limited, but biologically realistic, range of *h *examined, over which it has a relatively small effect on the function *σ *(see Methods). In addition, the outcome depends on the fitness of each genotype, which we consider in a separate section below.

### Sex determination transitions

Only two of the eight mutation pairs considered cause transitions in the sex determination locus from the D to the R locus: f→f^-^/a→A and m→m^+^/a→A (Figure 2(e) and 2(h), Table 3). Transitions occur when *k *is small (Eq (1)-(3)) and the A allele causes a large change in gene expression at the D locus. When this is sufficient for ${\hat{S}}_{\text{D}}$ to breach the *θ *threshold, it causes a change in genotypic sex (e.g. f^-^/f^- ^is male instead of female when down-regulated by AA). It can then lead to fixation of the regulated allele at the D locus (either f^- ^or m^+^), with transition of the control of sex determination to genetic variation at the R locus (Table 3). Transitions involving m^+ ^mutation pairs follow a similar pattern to those seen with the f^- ^mutation discussed in detail above (Figure 2(h)).

The remaining six mutation pairs never generate transitions. These are not possible when the a→A mutation precedes mutation at the D locus, simply because transitions require polymorphism at the R locus. In these cases, the A allele is assumed to have gone to fixation, so the R locus is monomorphic. We relaxed this constraint by allowing a "back" mutation at the R locus (from A→a, with output *Z*_a _= 0) following mutation at the D locus. Under these conditions we again observed sex determination locus transitions with the f^- ^and m^+ ^mutation pairs, but not with f^+ ^and m^- ^(Additional file 2). The range of *k *needed for the transitions follows the same pattern as seen with the f→f^-^/a→A and m→m^+^/a→A mutation pairs (Figure 2(e) and 2(h)).

A second constraint governs the absence of transitions for f→f^+^/a→A and m→m^-^/a→A. Consider f→f^+^/a→A. In order for a transition in the sex determination system to take place, all genotypes must evolve to be homozygous f^+ ^at the D locus (i.e. with loss of the m allele). However, as the A allele up-regulates f^+^, the output ${\hat{S}}_{\text{D}}$ for A genotypes will be at least as high as for the ancestral female, making a male f^+ ^homozygous genotype impossible. Similarly, for the m→m^-^/a→A mutation pair, the A allele down-regulates m^- ^and makes a female m^- ^homozygous genotype impossible. In these cases, transition of sex determination to the R locus cannot occur and build-up of the sex determination cascade is impossible [17], a condition close to the concept of "evolutionary constraint" (an unreachable region of evolutionary space [43]).

The two cases that allow sex determination transitions, f→f^-^/a→A and m→m^+^/a→A (Figure 2(e) and 2(h)), do not fall under either of these constraints. In both cases, polymorphism occurs at both the R and D loci and homozygotes of f^- ^and m^+ ^alleles can segregate in males and females.

### Recruitment and changes in heterogamety

Unlike transitions which are limited to two mutation pairs, recruitment events were observed for every mutational class (Table 3 and Figure 2). In a recruitment the mutant allele spreads but sex determination remains at the ancestral D locus. Recruitment occurs for all values of *k *and sometimes causes a change in heterogamety (Figure 2).

For high values of *k*, recruitment occurs without change in heterogamety (Figure 2, Table 3). In no case does the new regulatory interaction between the A and D loci cause ${\hat{S}}_{\text{D}}$ to breach *θ*, so no change in genotypic sex is possible. For example, in the a→A/m→m^+ ^mutation pair, the m^+ ^allele is up-regulated but is still a dominant masculinizer. Hence the A/A;m^+^/f genotype is male, like the ancestral A/A;m/f genotype. These simple replacements result in up- or down-regulation of expression at the D locus, but do not permit a change in heterogamety.

With lower values of *k*, recruitment can cause change from male to female heterogamety, which is seen for five of the eight mutation pairs (Figure 2). For example, in the a→A/f→f^+ ^mutation pair, the change in heterogamety occurs because the new f^+ ^allele is a dominant feminizer (A/A;m/f^+^), whereas in the a→A/f→f^- ^mutation pair, the new allele is a recessive masculinizer (A/A;f^-^/f^-^). The pattern is more complex with a→A/m→m^+ ^mutation pairs. In this case the up-regulation of m^+ ^caused by the A alleles is sufficient to cause ${\hat{S}}_{\text{D}}$ to breach *θ *and so result in sex change. There are three possibilities. Two of these cause a change in heterogamety: a dominant m^+ ^feminizer leading to A/A;m/m^+ ^females, or an m^+ ^allele which only feminizes in conjunction with the f allele leading to A/A;m^+^/f females. Alternatively the m^+ ^allele is a recessive feminizer leading to A/A;m^+^/m^+ ^females, which remain the homogametic sex. In contrast, changes in heterogamety never occur with the m^- ^mutation (either a→A/m→m^- ^or m→m^-^/a→A), as the m^- ^allele is a dominant masculinizer and so can never be expressed in a female.

Changes in heterogamety thus require the new regulatory connection to cause sufficient change in gene expression (${\hat{S}}_{\text{D}}$) towards the threshold for change in sex (*θ*) to result in a change in genotypic sex.

### Network fitness

Knowing the sex of each mutant genotype is not enough for predicting outcomes. These also depend on the fitness of each genotype, determined by *w*_M _and *w*_F_. These coefficients specify whether selection favors higher or lower expression at the D locus and scale the effect of changes in gene expression in males and females respectively.

The importance of selection is most easily seen when mutants only occur in one sex. Given ancestral male heterogamety, a few mutants only occur in males (Table 3), so their spread depends exclusively on *w*_M _(the value of *w*_F _is irrelevant). The condition for selection to favor spread is $\Delta {\hat{S}}_{\text{DM}}w_{\text{M}}>0$, causing an increase in male fitness, *W*_M _> 1. As there is no mutant female genotype, *W*_F _= 1. For example, in the mutation pair a→A/m→m^- ^(Figure 2(c)), the mutant A/A;m^-^/f is necessarily male as the m^- ^allele is down-regulated and $\Delta {\hat{S}}_{\text{DM}}<0$. Since no other genotypes are created, the only requirement for m^- ^to spread is that selection favors lower expression at the D locus in males (i.e. *w*_M _< 0). Male-only mutants (Table 3 column *W*_M _> 1, *W*_F _= 1) were found in all mutational classes, except for f→f^+^/a→A and a→A/f→f^+^. In these two cases, there are no conditions under which the f^+ ^mutation is ever restricted to males. There are no cases of female-only mutants (Table 3 column *W*_F _> 1, *W*_M _= 1). Under conditions of ancestral female heterogamety this pattern is reversed with female-only mutants but no male-only mutants (outcomes are symmetrical to ancestral male heterogamety, Additional file 1).

**Figure 3:**
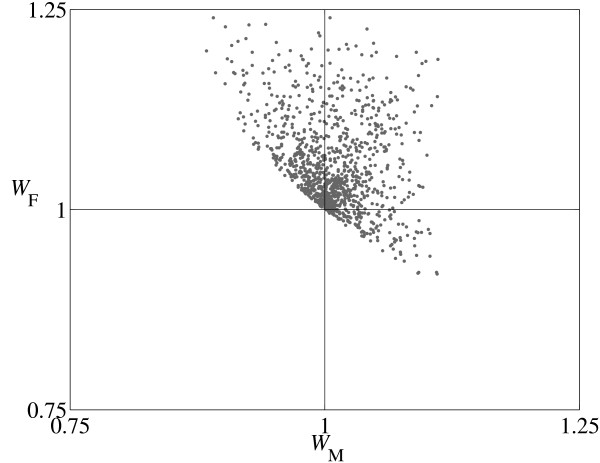
**Constraints on fitness**. The graph shows male (*W*_M_) and female (*W*_F_) fitness values for recruitment and transition events for the mutation pair a→A/f→f^- ^(Region III, Figure 2a). Note that both male-favoring (*W*_M _> 1, *W*_F _< 1) and female-favoring (*W*_M _< 1, *W*_F _> 1) sexual conflict are observed in this case. The global constraint (*W*_M _- 1) + (*W*_F _- 1) > 0 limits permissible values for recruitment.

When mutations occur in both sexes, they most frequently spread when selection favors them in both sexes (Table 3*, W*_M _> 1, *W*_F _> 1). However, there are many examples of sexual conflict in which the new regulatory connection is favored in one sex but deleterious in the other (i.e. *W*_M _> 1, *W*_F _< 1 or *W*_M _< 1, *W*_F _> 1). Consider the mutation pair a→A/f→f^- ^when *k *is large (Region III in Figure 2(a)). Here, a total of three new genotypes are created: one (m/f^-^) is male and the other two (f^-^/f and f^-^/f^-^) are female (note that all genotypes are homozygous A at the R locus). The new male genotype m/f^- ^has lower expression at the D locus (${\hat{S}}_{\text{D}}$) than the ancestral m/f male, and therefore $\Delta {\hat{S}}_{\text{DM}}<0$. Similarly the two mutant female genotypes (f^-^/f and f^-^/f^-^) also have lower ${\hat{S}}_{\text{D}}$ than the ancestral f/f female, so $\Delta {\hat{S}}_{\text{DF}}<0$ in both cases. When selection favors lower D expression in both sexes (*w*_M _< 0, *w*_F _< 0), the new genotypes raise fitness in both sexes (*W*_M _> 1, *W*_F _> 1). However when lower expression is only favored in one sex (either *w*_M _> 0, *w*_F _< 0 or *w*_M _< 0, *w*_F _> 0) there is sexual conflict (either *W*_M _< 1, *W*_F _> 1 or *W*_M _> 1, *W*_F _< 1 respectively). Under this condition, recruitment occurs as long as the combined fitness across the sexes is raised, (*W*_M _- 1)+(*W*_F _- 1) > 0 (Figure 3). The latter condition applies to all recruitment and transition events.

### Protected polymorphisms

**Figure 4:**
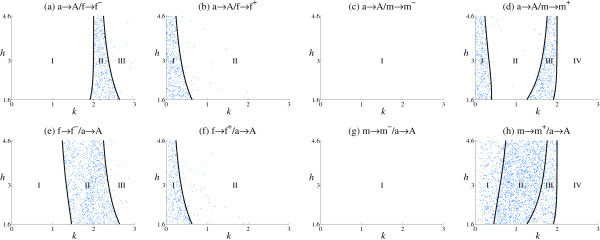
**Protected polymorphisms**. The distribution of mutations leading to protected polymorphisms, across the parameter space (*k *and *h*), is shown with curves delimiting each region as in Figure 2. Each point represents a particular simulation run that results in protected polymorphism.

Many mutations lead to protected polymorphisms in which there is more than one male or female genotype at equilibrium (Table 3, Figure 4). For a→A/X mutation pairs (i.e. the A regulatory allele already exists in the background state before a *cis *mutation occurs in the m or f alleles), polymorphism occurs at the D locus, while for X/a→A mutation pairs (i.e. *cis *mutation occurs in the m or f allele before mutation to the A allele), polymorphism occurs at both the A and D loci. Protected polymorphisms are restricted to those sub-regions (Figure 4) that otherwise cause a change in heterogamety or transitions (Figure 2). Under certain conditions, more than one genotype is generated in both sexes and persists at the polymorphic equilibrium.

**Figure 5:**
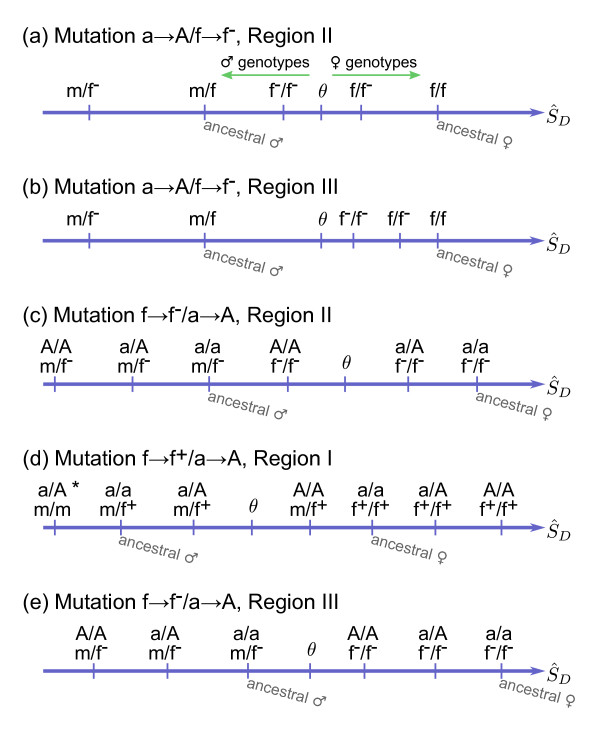
**D locus expression and sex**. In these examples, we illustrate how ${\hat{S}}_{\text{D}}$ defines sex for five different cases: (a) a→A/f→f^-^, Region II, (b) a→A/f→f^-^, Region III, (c) f→f^-^/a→A, Region II, (d) f→f^+^/a→A, Region I (* indicates this genotype could be a/A;m/m or A/A;m/m as both genotypes have the same ${\hat{S}}_{\text{D}}$ value), and (e) f→f^-^/a→A, Region III. In (a) and (b) the A allele is homozygous in all genotypes (not shown).

**Figure 6:**
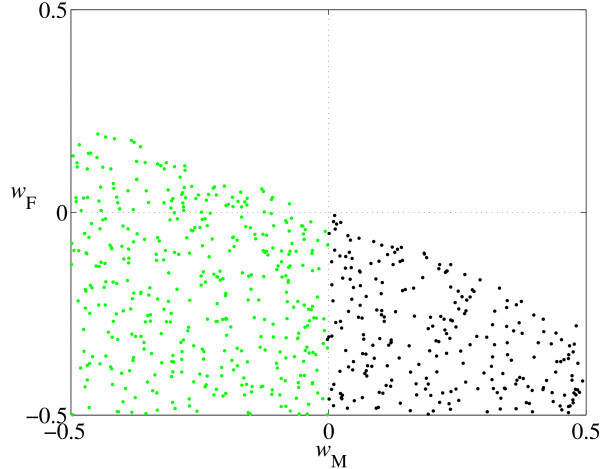
**Protected polymorphism and fitness**. The mutation pair a→A/f→f^- ^in Region II (Figure 5(a)) either leads to recruitment of f^- ^(black points) when selection favors higher expression in males (*w*_M _> 0) or protected polymorphism (green points) when selection favors lower expression in males (*w*_M _< 0).

As an example, consider Region II of mutation pair a→A/f→f^- ^(Table 3, Figure 4(a)). Here, there are two new male genotypes and one new female genotype (Figure 5(a)). Protected polymorphism typically occurs when selection favors lower expression in males *w*_M _< 0 and females *w*_F _< 0 (Figure 6 green points), so the f^- ^allele is favored in m/f^- ^males and f/f^- ^females, but not in f^-^/f^- ^males. This results in the retention of m, f and f^- ^alleles (Figure 5(a)). Protected polymorphism also occurs in a small region when *w*_M _< 0 and *W*_F _> 0 (Figure 6), where selection against the new female genotype (f/f^-^) is not sufficient to eliminate the new f^- ^allele that is favored in f^-^/f^- ^males alone.

Protected polymorphisms are abundant in some regions (Figure 4). With a→A/X mutation pairs, protected polymorphism occurs when selection favors retention of all three alleles at the D locus. This is the case when the ordering of ${\hat{S}}_{\text{D}}$ follows the pattern of Figure 5(a), as described above. With the alternative ${\hat{S}}_{\text{D}}$ ordering shown in Figure 5(b), protected polymorphism is rare or absent. Here, the novel female heterozygote (f^-^/f in this example) cannot be the most favored genotype as its expression lies between the new and ancestral homozygotes. So when selection favors lower expression in males and females (*w*_M _< 0, *w*_F _< 0), the new f^- ^allele is favored and goes to fixation. The same pattern is seen for f^+^, m^- ^and m^+ ^mutation pairs.

With X/a→A mutation pairs, protected polymorphisms require retention of multiple alleles across both the A and D loci. They are abundant in regions (Figure 4) in which selection can favor the R locus heterozygote in one sex and the D locus heterozygote in the other sex (an example of this pattern is given in Figure 5(c)). Protected polymorphism is also common if the double heterozygote is the most favored genotype in one sex (Figure 5(d)). In contrast, protected polymorphism is rare or absent from regions in which the expression of one of the single-locus heterozygotes lies between the new and ancestral homozygotes and so cannot be the most favored genotype (Figure 5(e)). These patterns are similar to those seen with a→A/X mutation pairs, but are more complex as they now involve two loci (for a list of ${\hat{S}}_{\text{D}}$ orderings for all mutation pairs see Additional file 3).

## Conclusion

In this paper, we develop a diploid gene network model that explicitly considers how *cis *and *trans *mutations contribute to evolutionary change in sex determination. Previous population genetic models [28-30,44] have ignored this and simply assumed each genotype is associated with a particular sex and fitness (e.g.1*+s*). Instead we let sex determination and fitness arise through the expression dynamics of the network. We then study evolution following a *cis *mutation in an existing sex determining allele (either m^+^, m^-^, f^+ ^or f^- ^alleles at the D locus) followed by a *trans *gain-of-function mutation at a locus not previously involved in sex determination (R locus) or *vice versa*.

Generally, we observe a many-to-many relationship as the same mutation can produce many outcomes and the same outcome can arise through many mutations (Figure 2, Table 3). In addition, the same outcome can result from many different selective regimes (e.g. mutants favored in both sexes or subject to sexual conflict, Table 3). These observations are important as they imply that changes in the sex determination systems of related species can arise in multiple ways, and a great deal of biological detail will be required to reconstruct the evolutionary history and selective regime that promoted evolutionary change [27].

Some constraints are identifiable. Of the eight possible mutation pairs (shown in Table 3), only two permit transitions in which a new upstream regulator takes over sex determination (f→f^-^/a→A and m→m^+^/a→A, in which the f^- ^and m^+ ^alleles exist prior to the mutation of a to A). In these cases, a single allele at the ancestral sex determination locus (D) is driven to fixation, with the A allele at the regulatory locus (R) becoming the new determiner of sex. For f→f^-^/a→A, the f^- ^allele goes to fixation. When the f^- ^homozygote is down-regulated by A it produces a male, but in the absence of A (or a lower dose of A) it is female (Figure 1, Table 3). Similarly for m→m^+^/a→A, the m^+ ^goes to fixation, and the m^+ ^homozygote is female when up-regulated by A, but male in the absence (or lower dose) of A. Such switching is not possible for mutations involving f^+ ^and m^- ^alleles. The up-regulation of f^+ ^and down-regulation of m^- ^homozygotes caused by the A regulator merely strengthens existing female and male signals respectively. Hence for these mutants, the ability to determine both male and female sexes requires retention of polymorphism at the ancestral D locus (i.e. f^+ ^and m^- ^mutant alleles cannot go to fixation), and sex determination cannot pass to the new R locus.

There is a further constraint as transitions to a new upstream regulator are only possible when mutation to f^- ^or m^+ ^*cis *alleles are established prior to the mutation of a to A *trans *regulator. Transitions are not possible when these mutations occur in the opposite order (Figure 2). This constraint arises for the simple reason that there must be polymorphism at the R locus for it to take over sex determination from the ancestral D locus. It can be overcome by allowing variance at the R locus through secondary "back" or null mutations from A to the a allele which lacks regulatory ability (Additional file 2). However, given that the A allele is likely to perform other regulatory functions prior to its recruitment for sex determination, "back" or null mutations are expected to be deleterious unless these other regulatory functions can be fulfilled in the hemizygous state or by other loci. This requirement is likely to restrict this evolutionary route. One way round this is duplication at the R locus, with retention of ancestral regulatory functions by one paralog and gain of the regulation of sex by the other paralog. Duplication appears to underlie recruitment of *Sxl *to the *Drosophila *sex determination network [45]. In the lineage leading to *Drosophila*, the ancestral form of *Sxl *underwent a duplication. One *Sxl *paralog gained a role in sex determination. The other paralog (*CG3056*) has retained the ancestral, non-sexual, regulatory functions as its expression pattern is similar to the non-duplicated version of *Sxl *found in the related fly *Megaselia scalaris *[45]. In terms of our model, this suggests a m→m^+^/a→A sequence of mutations, in which the novel *Sxl *allele (A allele at the R locus) was capable of up-regulating an ancestral m+ allele of *tra *(D locus). In this reconstruction we assume that the ancestral sex determination system was based on *tra *locus polymorphism [27].

As well as these network features, recruitment of mutants is most likely when they are favored in both sexes (*W*_F_, *W*_M _> 1), or are favored in one sex but do not segregate in the other (*W*_F _> 1, *W*_M _= 1 or *W*_F _= 1, *W*_M _> 1, Table 3). This requires that selection acts in the same direction as the change in expression caused by the new regulatory interaction ($W_{\text{F}}=1+\Delta {\hat{S}}_{\text{DF}}w_{\text{F}}$ for females and $W_{\text{M}}=1+\Delta {\hat{S}}_{\text{DM}}w_{\text{M}}$ for males). However, mutants causing sexual conflict, benefit in one sex and harm in the other (*W*_M _> 1, *W*_F _> 1 or *W*_M _< 1, *W*_F _> 1), spread with surprising frequency as well (Table 3). We found that for mutations to spread requires (*W*_M _- 1)+(*W*_F _- 1) > 0. That is, given the sex ratio is constrained to be 1:1, there must be an overall fitness gain summed across the mutant male and female genotypes, as has been pointed out in other contexts [28,30,46]. Our results suggest that transitions of sex determining systems and changes in heterogamety are not particularly dependent on sexually antagonistic selection [30,47].

Many mutations establish a new regulatory connection with the recruitment of new alleles but without change to a new sex determination locus (Table 3, Figure 2). Many of these amount to a simple replacement of an unregulated by a regulated allele at the D locus without change in genotypic sex or heterogamety (e.g. f^+ ^replaces f to give f^+^/f^+ ^females and m/f^+ ^males). Alternatively, the new regulatory arrangement causes a change in genotypic sex accompanied by a switch from male to female heterogamety (e.g. f^+ ^replaces f to give m/f^+ ^females and m/m males). The latter occurs when the effect of the upstream regulator A is relatively strong and causes a change in the sex of some genotypes. In either eventuality, A is fixed in the derived population. There are many potential examples of fixed regulators of sex determination genes. For example, the splicing activity of the SXL protein in *Drosophila *is dependent on interactions with a number of other proteins like SNF and PPS [48-50]. What remains to be determined is whether these interactions evolved before or after the involvement of *Sxl *in sex determination.

The conditions that give rise to changes in heterogamety can also result in protected polymorphisms in which more than one male or female genotype occurs at equilibrium (Figure 4). Protected polymorphisms occur when selection favors heterozygotes in both sexes at either the A or D loci (or both). It is surprising how easily protected polymorphisms arise in our model given that they are rare in nature, although they have been found in the housefly *Musca domestica *[28] and the platyfish *Xiphophorus maculates *[51]. These multilocus combinations give rise to some genotypes with low fitness, and so are probably prone to further evolutionary change, for example involving variations in sex ratio [28,52] or complex meta-population structures [53], that resolve them in favor of simpler systems with single male and female genotypes. In natural examples, other traits are associated with the sex determination alleles and selection on them maintains the polymorphism [28,51].

We have extensively studied evolutionary change when the ancestral condition is male heterogamety. Symmetrical constraints apply when the ancestral condition is female heterogamety. Here, transitions to a new sex determination locus only occur with the f→f^+^/a→A and m→m^-^/a→A mutation pairs (Additional file 1). Thus, change in the sex determination locus can occur for all four D locus alleles (f^+^, f^-^, m^+^, m^-^), but is dependent on ancestral heterogamety.

The importance of the model parameters *k *(and *h *to a lesser degree, Figure 2) in constraining network changes may shed light on the rate of turnover of sex determination systems. The parameter *k *determines the level of constitutive expression. It has a major effect on the evolutionary outcome. When *k *is high, changes in gene expression mediated by the regulatory locus (R) are relatively weak and thus major re-organisation of the sex determination systems does not occur. In effect this stabilizes the established sex determination mechanism. A high value of *k *might therefore explain the rareness of heterogamety changes in birds [54] and mammals [55]. In contrast, when *k *is small, regulation is relatively strong, opening up the possibility of changes in genotypic sex, transitions of the sex determination system and changes in heterogamety. The latter are common in frogs [56], medaka fish [57] and insects [58,59]. Differential regulation is clearly not the only possible force underlying changes in sex determination systems. Alternative explanations such as sexually antagonistic selection [30,47] and sex ratio bias [28] are likely to impose different sets of constraints.

The work in this paper was motivated by the complexities of the sex determination mechanism of *Drosophila melanogaster *and related species [12,13]. We have shown the importance of explicitly considering changes in network dynamics, due to *cis *and *trans *mutations, for predicting transitions to a new upstream sex determination locus as well as the recruitment of other regulators which establish new regulatory interactions at sex determining loci. Our network approach allows sex and fitness to emerge directly from the regulatory interactions rather than being imposed by assumptions made about "modifiers". This approach demonstrates that the same mutation can produce many different evolutionary outcomes, while the same evolved state can be produced by many different mutations.

## Methods

We use a standard population genetics simulation to study the evolutionary fate of each mutation. The model assumes an infinite diploid population with non-overlapping generations, with a 1:1 (male:female) sex ratio. Each mutant allele was introduced at low frequency (0.5%) in the heterogametic sex (results obtained for mutants in the homogametic sex were qualitatively equivalent). At each generation the following steps were taken. First, gene expression ${\hat{S}}_{\text{D}}$ was calculated for each genotype, followed by sex determination. Adult male and female genotype frequencies were calculated using the fitness values (*W*_M_, *W*_F_) of each genotype. Random mating amongst adults assuming unlinked loci was used to define the zygote frequencies in the following generation. This procedure was repeated until the population reached equilibrium. We measured the maximum difference (over the set of all genotypes, G) between successive timesteps, *e*(*t*) = max_G_|*p*(*t*) - *p*(*t *- 1)|, where *p*(*t*) is the frequency of a genotype at generation *t*. The system is considered stable when *e*(*t*) < 10^-12 ^. We imposed a maximum of 10^11 ^timesteps, however, in every simulation the criterion was reached before this maximum number of steps. A mutant allele was classified as non-invasive if it was driven out, with the final population being the same as the ancestral population. Invasive mutants resulting in recruitments or transitions caused the loss of one of the ancestral alleles. To distinguish invasive mutants resulting in protected polymorphism from neutral or very weakly selected mutants, we first removed genotypes at ≤1% frequency (since mutants are inserted at 0.5% allele frequency, 1% is the upper bound genotype frequency for neutral mutants). If more than one female or male genotype was observed (i.e. protected polymorphism), a second genotype threshold of 0.1% was used to ensure inclusion of all relevant genotypes. Simulations were performed using a custom-built C++ program capable of generating novel genotypes in real time and assigning sex and fitness dynamically. Examples of genotype frequency dynamics for simulations leading to both recruitment and protected polymorphism are shown in Additional file 4.

For each of the eight network mutations, 10,000 independent evolutionary simulations were performed, with a set of random parameter values (for *h*, *k*, *w*_M_, and *w*_F_) which remain fixed for the entire duration of each simulation. A uniform distribution is assumed for all parameters. For *h*, we assume the biologically realistic range (1.6,4.6) observed for a wide range of behavior *in vitro *in the *lac *operon (Table 1 and [60]). For *k*, we adopted the range (0,3), since no interesting behavior is observed for higher values (see Figure 2). For both *w*_M _and *w*_F _we use the range (-1/2,1/2) to avoid negative fitness values [the range for $\Delta {\hat{S}}_{\text{D}}$ is (-2,+2)]. The sex determination threshold *θ *is calculated once *h *and *k *are known and remains fixed throughout the simulation. Separate experiments were performed for different strengths of the regulatory allele A (i.e. *T*_A _= -1 or +1). In the main text we only report the case *T*_A _= +1 (i.e. high constitutive expression) as the other case is qualitatively equivalent; see Additional file 5 (*T*_A_= -1).

## Authors' contributions

All authors conceived the study and contributed to the writing of the manuscript. TM performed the simulations and analyzed the results. All authors read and approved the final manuscript

## Supplementary Material

Additional file 1 **Symmetry between ancestral male and female heterogamety**. We demonstrate that the results reported in the text for **male **heterogamety are symmetric to those found with ancestral **female **heterogamety. Click here for file

Additional file 2 **Back mutations at the R-locus**. Transitions are possible with the mutation pairs a→A/f→f^- ^or a→A/m→m^+ ^if back mutations are permitted to generate polymorphism at the R locus (i.e. A→a). Click here for file

Additional file 3 **Genotype ordering with respect to **${\hat{S}}_{\text{D}}$. The information in this file shows the value of ${\hat{S}}_{\text{D}}$ for all mutation pairs and regions. A selection of these are illustrated in Figure 5. Click here for file

Additional file 4 **Simulation examples**. Per generation change in genotype frequency is shown for two cases, one leading to recruitment, the other to protected polymorphism. Click here for file

Additional file 5 **Simulation results with T_A _= -1**. In the text we consider the case of high constitutive expression of the A allele (i.e. T_A _= +1). We demonstrate qualitatively similar results with low constitutive expression of the A allele (i.e. T_A _= -1). Click here for file
