# Supplementary material for: Differential regulation drives plasticity in sex determination gene networks
Source: BMC Evol Biol. 2010 Dec 16;10:388. doi: 10.1186/1471-2148-10-388 (PMC3022605; doi:10.1186/1471-2148-10-388)
Supplement: Additional file 1 — Symmetry between ancestral male and female heterogamety. We demonstrate that the results reported in the text for male heterogamety are symmetric to those found with ancestral female heterogamety. [file 1471-2148-10-388-S1.PDF]

Additional file 1

Symmetry between ancestral male and female heterogamety

As shown in the main text, under ancestral male heterogamety $\theta = \sigma(k) + 1/2$ and sex is determined by the sign of

$$g = \hat{S}_{\mathrm{D}} - \theta = \hat{S}_{\mathrm{D}} - [\sigma(k) + 1/2] \ .$$

We can represent the form of any genotype as

$$g_{\mathrm{m}} = \sigma(\alpha_{\mathrm{d1}}\sigma(kT_{\mathrm{A}}) + kT_{\mathrm{d1}}) + \sigma(\alpha_{\mathrm{d2}}\sigma(kT_{\mathrm{A}}) + kT_{\mathrm{d2}}) - \sigma(k) - 1/2$$

where $\alpha_i \in \{-2,-1,0,1,2\}$ represents the input term $\sum_j I_{i,j} Z_j$ from equation (3) in the main text and indicates the number ($|\alpha_i|$) of A locus inputs, and their sign effect (negative for m^- and f^-, positive for m^+, f^+). The first term corresponds to the output for allele d1, and the second term to the output for allele d2.

Ancestral female heterogamety is symmetric to this if we apply the transformation: a) interchange the alleles m and f, and b) the sign of their input from the R locus. For example, the mutation $f \rightarrow f^-$/a→A under ancestral male heterogamety is symmetric to the mutation $m \rightarrow m^+$/a→A under ancestral female heterogamety. This is equivalent to changing the sign of both $\alpha_{\mathrm{d}i}$ and $T_{\mathrm{d}i}$. We can then define g_{f}, the form of *g* for ancestral female heterogamety, given that $\theta = \sigma(-k) + 1/2 = 3/2 - \sigma(k)$,

$$g_{\mathrm{f}} = \sigma(-\alpha_{\mathrm{d1}}\sigma(kT_{\mathrm{A}}) - kT_{\mathrm{d1}}) + \sigma(-\alpha_{\mathrm{d2}}\sigma(kT_{\mathrm{A}}) - kT_{\mathrm{d2}}) - [3/2 - \sigma(k)] .$$

Since $\sigma(-x) = 1 - \sigma(x)$,

$$g_{\mathrm{f}} = -\sigma(\alpha_{\mathrm{d1}}\sigma(kT_{\mathrm{A}}) + kT_{\mathrm{d1}}) - \sigma(\alpha_{\mathrm{d2}}\sigma(kT_{\mathrm{A}}) + kT_{\mathrm{d2}}) + \sigma(k) + 1/2 = -g_{\mathrm{m}}$$

Thus, after the transformation, sex is simply reversed (female ↔ male) for each genotype with respect to its pre-transformation equivalent.

In a similar way, symmetry also arises for fitness ($W_{\mathrm{M}}, W_{\mathrm{F}}$), which depends on $\Delta\hat{S}_{\mathrm{D}}$. Under ancestral male heterogamety (AMH), males have ancestral expression $\hat{S}_{\mathrm{D}} = \hat{S}_{\mathrm{m}} + \hat{S}_{\mathrm{f}} = \sigma(kT_A) + \sigma(-kT_A) = 1$. Therefore,

$$\Delta\hat{S}_{\mathrm{D(AMH)}} = \sigma(\alpha_{\mathrm{d1}}\sigma(kT_{\mathrm{A}}) + kT_{\mathrm{d1}}) + \sigma(\alpha_{\mathrm{d2}}\sigma(kT_{\mathrm{A}}) + kT_{\mathrm{d2}}) - 1$$

Under ancestral female heterogamety (AFH), females are m/f and also have ancestral expression $\hat{S}_{\mathrm{D}} = 1$.

Applying the transformation here, we have

$$\Delta\hat{S}_{\mathrm{D(AFH)}} = \sigma(-\alpha_{\mathrm{d1}}\sigma(kT_{\mathrm{A}}) - kT_{\mathrm{d1}}) + \sigma(-\alpha_{\mathrm{d2}}\sigma(kT_{\mathrm{A}}) - kT_{\mathrm{d2}}) - 1$$

$$\Delta\hat{S}_{\mathrm{D(AFH)}} = -\sigma(\alpha_{\mathrm{d1}}\sigma(kT_{\mathrm{A}}) + kT_{\mathrm{d1}}) - \sigma(\alpha_{\mathrm{d2}}\sigma(kT_{\mathrm{A}}) + kT_{\mathrm{d2}}) + 1 = -\Delta\hat{S}_{\mathrm{D(AMH)}}$$

Now, under ancestral male heterogamety, females are f/f and have ancestral expression $\hat{S}_{\mathrm{D}} = 2\sigma(k)$.

Therefore,

$$\Delta\hat{S}_{\mathrm{D(AMH)}} = \sigma(\alpha_{\mathrm{d1}}\sigma(T_{\mathrm{A}}k) + kT_{\mathrm{d1}}) + \sigma(\alpha_{\mathrm{d2}}\sigma(T_{\mathrm{A}}k) + kT_{\mathrm{d2}}) - 2\sigma(k)$$

Under ancestral female heterogamety, males are m/m and have ancestral expression $\hat{S}_{\mathrm{D}} = 2\sigma(-k)$. Applying the transformation, we have

$$\Delta\hat{S}_{\mathrm{D(AFH)}} = \sigma(-\alpha_{\mathrm{d1}}\sigma(kT_{\mathrm{A}}) - kT_{\mathrm{d1}}) + \sigma(-\alpha_{\mathrm{d2}}\sigma(kT_{\mathrm{A}}) - kT_{\mathrm{d2}}) - 2\sigma(-k)$$

$$\Delta\hat{S}_{\mathrm{D(AFH)}} = -\sigma(\alpha_{\mathrm{d1}}\sigma(kT_{\mathrm{A}}) + kT_{\mathrm{d1}}) - \sigma(\alpha_{\mathrm{d2}}\sigma(kT_{\mathrm{A}}) + kT_{\mathrm{d2}}) + 2\sigma(k) = -\Delta\hat{S}_{\mathrm{D(AMH)}}$$

Therefore, to extend the symmetric transformation to include fitness, we simply interchange w_{M} and w_{F}, changing the sign on both. This will cause an interchange $W_{\mathrm{M}} \leftrightarrow W_{\mathrm{F}}$ after the transformation.
