# Supplementary material for: Differential regulation drives plasticity in sex determination gene networks
Source: BMC Evol Biol. 2010 Dec 16;10:388. doi: 10.1186/1471-2148-10-388 (PMC3022605; doi:10.1186/1471-2148-10-388)
Supplement: Additional file 2 — Back mutations at the R-locus. Transitions are possible with the mutation pairs a→A/f→f- or a→A/m→m+ if back mutations are permitted to generate polymorphism at the R locus (i.e. A→a). [file 1471-2148-10-388-S2.PDF]

Additional File 2

Back mutations at the R-locus

Transitions in the sex determination system cannot take place for the mutation pairs a→A/f→f^- and a→A/m→m^+ because the A allele is assumed to have gone to fixation before the f^- or m^+ mutations are introduced. However, transitions can occur with back mutation from A→a (Table Additional File 2). Transitions occur in Region I for the mutation pair a→A/f→f^-, and in Region II for the mutation pair a→A/m→m^+ (Figure 2). Back mutations were introduced into either the male or female genotype. However there was no difference in the result (counts are shown for mutation in the male genotype). No transitions were recorded with a→A/f→f^+, a→A/m→m^-, f→f^+/a→A or m→m^-/a→A mutation pairs.

Table Additional File 2: Transition frequencies with back mutation from allele A→a.

	Intermediate genotypes		Evolved genotypes		
1st Mutation Pair	Male	Female	Male	Female	Transitions
a→A/f→f^-	A/A;f/f^-	A/A;f/f	a/A;f^-/f^-	a/a;f^-/f^-	40
	A/A;f/f^-	A/A;f/f	A/A;f^-/f^-	a/A;f^-/f^-	41
a→A/m→m^+	A/A;m/m^+	A/A;m^+/m^+	a/A;m^+/m^+	A/A;m^+/m^+	1025
