# Supplementary material for: Differential regulation drives plasticity in sex determination gene networks
Source: BMC Evol Biol. 2010 Dec 16;10:388. doi: 10.1186/1471-2148-10-388 (PMC3022605; doi:10.1186/1471-2148-10-388)
Supplement: Additional file 3 — Genotype ordering with respect to S^D. The information in this file shows the value of S^D for all mutation pairs and regions. A selection of these are illustrated in Figure 5. [file 1471-2148-10-388-S3.PDF]

Additional file 3

Genotype ordering with respect to $\hat{S}_\mathrm{D}$

Below we detail the ordering of genotypes for $\hat{S}_\mathrm{D}$ for all mutation pairs and regions (some of these are illustrated in Figure 5). For each mutation/region combination we show male and female genotypes (separated by θ). In most regions, multiple orderings are possible and these are shown on separate lines. In two regions (f→f^+/a→A Region 1 and m→m^+/a→A Region 2) there are differences with respect to the sex of some genotypes, but the outcome in terms of transitions, recruitment and protected polymorphism is the same (these subregions are labeled as -A, -B). Some regions contain multiple genotypes with identical $\hat{S}_\mathrm{D}$ values (e.g. genotypes a/a;m/m and a/A;m/m). Genotypes for a→A/X mutations are shown without the R locus alleles, as they are always homozygous A/A.

1. a→A/X mutations

1.1. Mutation a→A/f→f^-

1.1.1 a→A/f→f^- Region 1

m/f-   m/f   f-/f   θ   f/f

m/f-   f-/f   m/f   θ   f/f

1.1.2. a→A/f→f^- Region 2

m/f-   m/f   f-/f-   θ   f-/f   f/f

1.1.3. a→A/f→f^- Region 3

m/f-   m/f   f-/f-   θ   f-/f   f/f

1.2. Mutation a→A/f→f^+

1.2.1 a→A/f→f^+ Region 1

m/m m/f θ f/f m/f+ f+/f

m/m m/f θ m/f+ f/f f+/f

1.2.2 a→A/f→f^+ Region 2

m/f m/f+ θ f/f f+/f f+/f+

1.3. Mutation a→A/m→m^-

1.3.1 a→A/m→m^- Region 1

m−/f m/f θ f/f

1.4. Mutation a→A/m→m^+

1.4.1 a→A/m→m^+ Region 1

m/m m/f θ f/f m+/m m+/f

m/m m/f θ m+/m f/f m+/f

1.4.2 a→A/m→m^+ Region 2

m/f m+/m θ f/f m+/f m+/m+

m+/m m/f θ m+/m+ m+/f f/f

1.4.3 a→A/m→m^+ Region 3

m+/m m/f m+/m+ θ m+/f f/f

1.4.4 a→A/m→m^{+} Region 4

m/f m+/f θ f/f

2. X/a→A mutations

2.1. Mutation f→f^{-}/a→A

2.1.1 f→f^{-}/a→A Region 1

a/A;m/f- a/a;m/f- a/A;f-/f- θ a/a;f-/f-

a/A;m/f- a/A;f-/f- a/a;m/f- θ a/a;f-/f-

a/A;f-/f- a/A;m/f- a/a;m/f- θ a/a;f-/f-

2.1.2 f→f^{-}/a→A Region 2

A/A;m/f- a/A;m/f- a/a;m/f- A/A;f-/f- θ a/A;f-/f- a/a;f-/f-

A/A;m/f- a/A;m/f- A/A;f-/f- a/a;m/f- θ a/A;f-/f- a/a;f-/f-

A/A;m/f- A/A;f-/f- a/A;m/f- a/a;m/f- θ a/A;f-/f- a/a;f-/f-

2.1.3 f→f^{-}/a→A Region 3

A/A;m/f- a/A;m/f- a/a;m/f- θ A/A;f-/f- a/A;f-/f- a/a;f-/f-

2.2. Mutation f→f^{+}/a→A

2.2.1-A f→f^{+}/a→A Region 1-A (Contains neutral males)

a/a;m/m a/A;m/m A/A;m/m a/a;m/f+ θ a/a;f+/f+ a/A;m/f+ A/A;m/f+ a/A;f+/f+

a/a;m/m a/A;m/m A/A;m/m a/a;m/f+ θ a/A;m/f+ a/a;f+/f+ A/A;m/f+ a/A;f+/f+

a/a;m/m a/A;m/m A/A;m/m a/a;m/f+ θ a/A;m/f+ A/A;m/f+ a/a;f+/f+ a/A;f+/f+

2.2.1-B f→f^{+}/a→A Region 1-B (Contains neutral males)

a/A;m/m A/A;m/m a/a;m/f+ a/A;m/f+ θ A/A;m/f+ a/a;f+/f+ a/A;f+/f+ A/A;f+/f+

2.2.2 f→f^+/a→A Region 2

a/a;m/f+ a/A;m/f+ A/A;m/f+   θ   a/a;f+/f+ a/A;f+/f+ A/A;f+/f+

2.3. Mutation m→m^-/a→A

2.3.1 m→m^-/a→A Region 1 (Contains neutral females)

A/A;m−/f a/A;m−/f a/a;m−/f   θ   a/a;f/f a/A;f/f A/A;f/f

2.4. Mutation m→m^+/a→A

2.4.1 m→m^+/a→A Region 1 (Contains neutral females)

a/a;m+/m+ a/a;m+/f   θ   a/a;f/f   a/A;f/f   a/A;m+/f a/A;m+/m+

a/a;m+/m+ a/a;m+/f   θ   a/A;m+/m+ a/A;m+/f a/a;f/f   a/A;f/f

2.4.2A m→m^+/a→A Region 2-A (Contains neutral females)

a/a;m+/m+ a/a;m+/f a/A;m+/m+   θ   a/A;m+/f a/a;f/f a/A;f/f A/A;m+/f A/A;m+/m+

2.4.2B m→m^+/a→A Region 2-B (Contains neutral females)

a/A;m+/m+ a/a;m+/f a/A;m+/f   θ   A/A;m+/m+ A/A;m+/f a/a;f/f a/A;f/f A/A;f/f

2.4.3 m→m^+/a→A Region 3 (Contains neutral females)

a/A;m+/m+ a/a;m+/f a/A;m+/f   A/A;m+/m+   θ   A/A;m+/f a/a;f/f a/A;f/f A/A;f/f

a/A;m+/m+ a/a;m+/f A/A;m+/m+ a/A;m+/f   θ   A/A;m+/f a/a;f/f a/A;f/f A/A;f/f

2.4.4 m→m^+/a→A Region 4 (Contains neutral females)

a/a;m+/f a/A;m+/f A/A;m+/f   θ   a/a;f/f a/A;f/f A/A;f/f
