# Supplementary material for: Differential regulation drives plasticity in sex determination gene networks
Source: BMC Evol Biol. 2010 Dec 16;10:388. doi: 10.1186/1471-2148-10-388 (PMC3022605; doi:10.1186/1471-2148-10-388)
Supplement: Additional file 4 — Simulation examples. Per generation change in genotype frequency is shown for two cases, one leading to recruitment, the other to protected polymorphism. [file 1471-2148-10-388-S4.PDF]

Additional file 4

Simulation examples

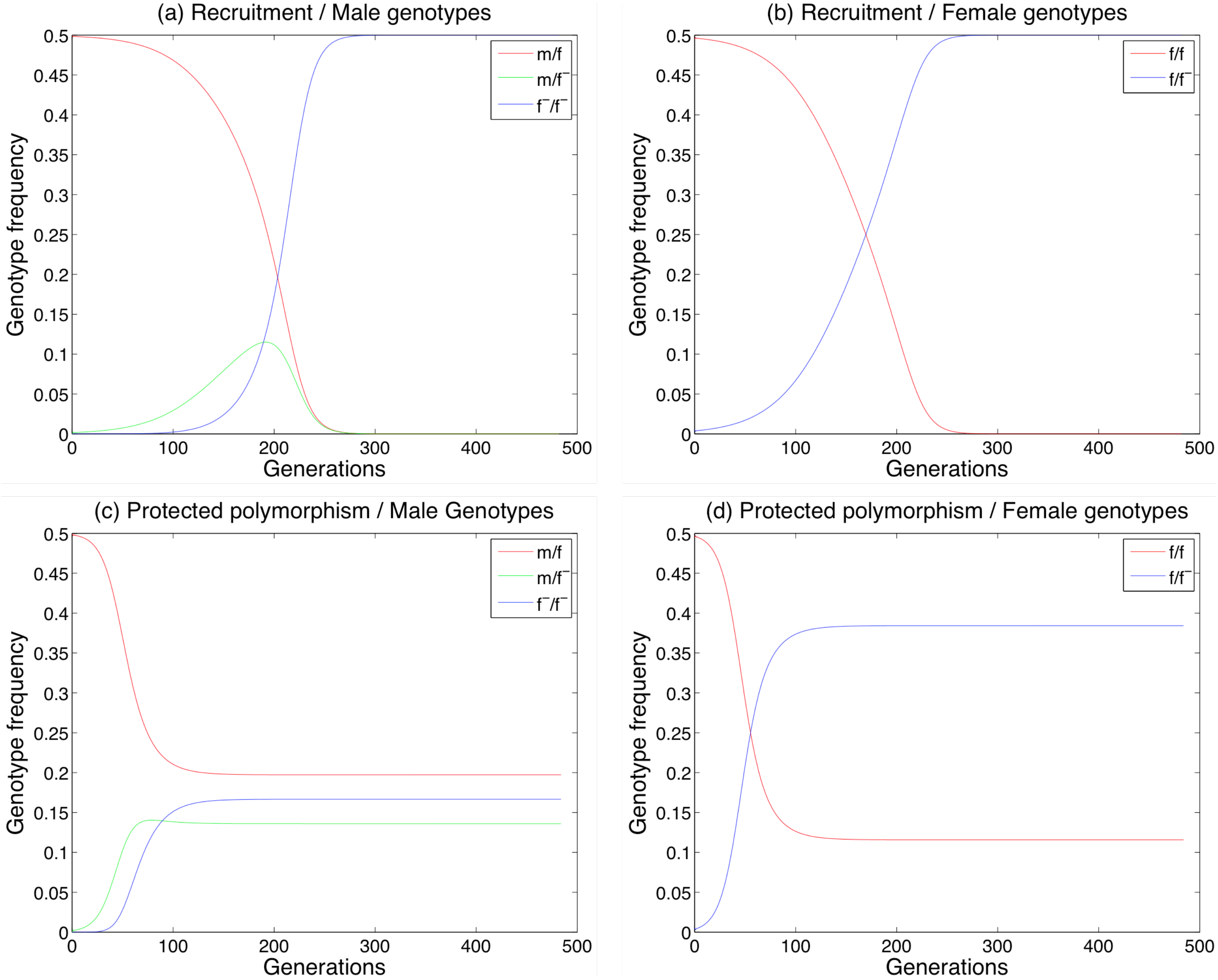

Time progression of genotype frequencies for the mutation pair a→A/f→f¯ in Region II (Figure 2). Parameter values were set to *k*=2.1 and *h*=3, with fitness $w_M = 0.25$ and $w_F = -0.25$ (a) and (b), and in contrast $w_M = -0.25$, $w_F = -0.25$ for (c) and (d).
