# Supplementary material for: Differential regulation drives plasticity in sex determination gene networks
Source: BMC Evol Biol. 2010 Dec 16;10:388. doi: 10.1186/1471-2148-10-388 (PMC3022605; doi:10.1186/1471-2148-10-388)
Supplement: Additional file 5 — Simulation results with TA = -1. In the text we consider the case of high constitutive expression of the A allele (i.e. TA = +1). We demonstrate qualitatively similar results with low constitutive expression of the A allele (i.e. TA = -1). [file 1471-2148-10-388-S5.PDF]

Additional file 5

Simulation results with $T_A = -1$

			Evolved genotype		Recruitment/transition selection					
**Mutation pair**	**Region**	**Outcome**	**Male**	**Female**	$W_M>1$ $W_F=1$	$W_M=1$ $W_F>1$	$W_M>1$ $W_F>1$	$W_M>1$ $W_F<1$	$W_M<1$ $W_F>1$	**Protected polymorphism**
a→A/f→f^-	I	R	f/f^-	f/f	698	0	0	0	0	0
	II	RH	f^-/f^-	f/f^-	0	0	90	0	1	127
	III	R	m/f^-	f^-/f^-	0	0	1850	475	1369	6
a→A/f→f^+	I	RH	m/m	m/f^+	0	0	136	57	2	341
	II	R	m/f^+	f^+/f^+	0	0	2016	483	1538	6
a→A/m→m^-	I	R	m^-/f	f/f	4715	0	0	0	0	0
a→A/m→m^+	I	RH	m/m	m/m^+	0	0	112	27	4	220
	II	R	m/m^+	m^+/m^+	0	0	103	27	79	1
	III	RH	m^+/m^+	m^+/f	0	0	49	0	0	81
	IV	R	m^+/f	f/f	3927	0	0	0	0	0
f→f^-/a→A	I	T	a/A;f^-/f^-	a/a;f^-/f^-	749	0	0	0	0	0
	II	TH	A/A;f^-/f^-	a/A;f^-/f^-	0	0	72	0	10	151
	III	R	A/A;m/f^-	A/A;f^-/f^-	0	0	1881	420	1355	15
f→f^+/a→A	I	RH	A/A;m/m	A/A;m/f^+	0	0	179	46	59	273
	II	R	A/A;m/f^+	A/A;f^+/f^+	0	0	2022	502	1507	9
m→m^-/a→A	I	R	A/A;m^-/f	A/A;f/f	4498	0	0	0	0	0
m→m^+/a→A	I	TH	a/a;m^+/m^+	a/A;m^+/m^+	0	0	173	1	82	94
	II	T	a/A;m^+/m^+	A/A;m^+/m^+	0	0	1	1	6	196
	III	RH	A/A;m^+/m^+	A/A;m^+/f	0	0	66	3	1	59
	IV	R	A/A;m^+/f	A/A;f/f	3801	0	0	0	0	0

Note that the regions here are comparable to $T_A = +1$ in terms of outcome and relative position in parameter space (Table 2), but that the borders are not in exactly the same positions across the range of k and h. Due to computational constraints we used a slightly lower threshold of $e(t) < 10^{-11}$ for the population dynamics (see Methods).
